# Oxypeucedanin relieves LPS-induced acute lung injury by inhibiting the inflammation and maintaining the integrity of the lung air-blood barrier

**DOI:** 10.18632/aging.204235

**Published:** 2022-08-18

**Authors:** Li Du, Jinrong Zhang, Xiyue Zhang, Chunyan Li, Qi Wang, Guangping Meng, Xingchi Kan, Jie Zhang, Yuxi Jia

**Affiliations:** 1 Department of Respiratory and Critical Care Medicine, The Second Hospital of Jilin University, Changchun, Jilin, China; 2 Department of Pathogeny Biology, College of Basic Medical Sciences, Jilin University, Changchun, Jilin, China; 3 Department of Theoretic Veterinary Medicine, College of Veterinary Medicine, Jilin University, Changchun, Jilin, China; 4 Department of Orthopedics, The Second Hospital of Jilin University, Changchun, Jilin, China; 5 Application Demonstration Center of Precision Medicine Molecular Diagnosis, The Second Hospital of Jilin University, Changchun, Jilin, China

**Keywords:** acute lung injury, lipopolysaccharide, PI3K/AKT/NF-κB, MAPKs, molecular docking simulation

## Abstract

Introduction: Acute lung injury (ALI) is commonly accompanied by a severe inflammatory reaction process, and effectively managing inflammatory reactions is an important therapeutic approach for alleviating ALI. Macrophages play an important role in the inflammatory response, and this role is proinflammatory in the early stages of inflammation and anti-inflammatory in the late stages. Oxypeucedanin is a natural product with a wide range of pharmacological functions. This study aimed to determine the effect of oxypeucedanin on lipopolysaccharide (LPS)-induced ALI.

Methods and Results: In this study, the following experiments were performed based on LPS-induced models *in vivo* and *in vitro*. Using myeloperoxidase activity measurement, ELISA, qRT-PCR, and Western blotting, we found that oxypeucedanin modulated the activity of myeloperoxidase and decreased the expression levels of inflammatory mediators such as TNF-α, IL-6, IL-1β, MPO, COX-2 and iNOS in LPS-induced inflammation models. Meanwhile, oxypeucedanin inhibited the activation of PI3K/AKT and its downstream NF-κB and MAPK signaling pathways. In addition, oxypeucedanin significantly decreased the pulmonary vascular permeability, which was induced by LPSs, and the enhanced expression of tight junction proteins (Occludin and Claudin 3).

Conclusions: In conclusion, this study demonstrated that the anti-inflammatory mechanism of oxypeucedanin is associated with the inhibition of the activation of PI3K/AKT/NF-κB and MAPK signaling pathways and the maintenance of the integrity of the lung air-blood barrier.

## INTRODUCTION

Acute lung injury (ALI)/acute respiratory distress syndrome (ARDS) is a serious respiratory disease with symptoms such as acute attacks, progressive hypoxemia, and severe noncardiogenic pulmonary edema [1, 2]. ALI/ARDS has a high mortality rate, which can be as high as 40%, and has a large negative impact on public health [3, 4]. In the process of ALI/ARDS, acute pulmonary inflammation, alveolar and pulmonary interstitial edema, and destruction of the lung air-blood barrier aggravate the pathological process of ALI/ARDS [5, 6]. Current studies have shown that effectively controlling the inflammatory response in the process of ALI/ARDS is a potential treatment to alleviate the disease. However, there is no specific drug available. In particular, coronavirus disease 2019 (COVID-19) has ravaged the world and become a global pandemic and a public health crisis. Traditional Chinese medicine has played a positive role in treating the disease [7]. Therefore, studying the pharmacology of herbal medicine may positively affect COVID-19.

Macrophages play an important role in the inflammatory response. In the alveoli, macrophages are the first line of defense against microscopic particles and microorganisms. It has been shown that macrophages perform distinct and unique functions in different stages of ALI/ARDS [8, 9]. Regulating macrophages may be a potential approach for treating ALI/ARDS [10]. During the acute phase of ALI/ARDS, resident alveolar macrophages usually transition from expressing an alternatively activated phenotype (M2) to a classically activated phenotype (M1) and release various proinflammatory mediators, causing a severe inflammatory response [11]. During the repair phase of ALI/ARDS, resident and recruited macrophages shift from the M1 phenotype back to the M2 phenotype to eliminate apoptotic cells and activate anti-inflammatory signals [12, 13]. Lipopolysaccharides (LPSs) function as a common stimulus that triggers inflammation. *In vivo* and *in vitro* experiments involve many inflammation models and select clinical drug candidates based on the inflammatory response induced by LPS [14–16]. Therefore, LPS-induced mice and RAW264.7 cells are scientific and reasonable choices for the basis of this experiment. MAPKs and NF-κB are two classic pro-inflammatory signaling pathways, and the phosphorylation of MAPKs and NF-κB that LPS can activate can promote the secretion of inflammatory mediators, exacerbating the severity of ALI. Some studies have shown that inhibiting MAPKs and NF-κB may be a potential therapeutic strategy for alleviating ALI [17–19]. However, it has not been reported whether Oxypeucedanin (OPD) can inhibit the activation of MAPKs and NF-κB in an LPS-induced ALI model.

The lung air-blood barrier, or alveolar-capillary barrier, is an important physiological barrier. The main components of the lung air-blood barrier are lung epithelial cells, pulmonary capillary endothelial cells, and the tight junctions among the cells [20]. The lung air-blood barrier has important biological functions, such as protecting the invasion of pathogenic bacteria and maintaining the normal physiological metabolism of the lungs. The destruction of the lung air-blood barrier due to pathogenic microorganisms or mechanical damage leads to changes in the lung air-blood barrier’s permeability, which aggravates lung tissue edema and inflammatory infiltration [19, 20]. In contrast, when ALI-induced defects occur in the pulmonary gas and blood barrier functions, dysfunction of the pulmonary microvascular endothelial barrier is more important because it is the first barrier for substances such as fluids, proteins, and inflammatory mediators to enter the alveolar cavity from blood vessels [21]. Current studies have shown that protecting the integrity of the lung air-blood barrier is beneficial to alleviating ALI [21]. However, the role of OPD in protecting the lung barrier has not been reported.

OPD, one of the main active components of Angelica dahurica coumarin [22, 23], has a wide range of biological activities, such as anti-inflammatory, antioxidant, and antitumor activities [23, 24]. Due to its therapeutic value, Angelica dahurica, also known as “Baizhi”, has long been used in analgesia and influenza treatments and for other related inflammatory diseases.

In an *in vitro* experiment, OPD was reported to have a good anti-inflammatory effect in RBL-2H3 cells [25]. However, it has not been reported whether OPD can alleviate LPS-induced ALI. Therefore, the purpose of this study was to explore the effect of OPD on LPS-induced ALI and its potential mechanism to provide a theoretical basis for the treatment of ALI/ARDS.

## MATERIALS AND METHODS

### Materials

DMEM (SH30285. FS), 0.05% trypsin (SH30236.01), PBS solution (SH30256.01), fetal bovine serum (SV30087.03), penicillin, and streptomycin (SV30087.03) were purchased from HyClone Laboratories (Logan, UT, USA). TRIzol (93289), DMSO (D2650), LPS (L-2880), Evans Blue (E2129), and formamide (F7503) were obtained from Sigma–Aldrich (Saint Louis, MO, USA). Primary antibodies including ERK1/2 (9102), p-ERK1/2 (9101), p-AKT (4060), AKT (4691), p-PI3K (4228), PI3K (4257), p38 (9212), p-P38 (9211), NF-κB p65 (4764), NF-κB p-p65 (3033), JNK (9252), p-JNK (4668), β-actin (3700), cyclooxygenase-2 (ab62331), iNOS (ab178945), Occludin (ab216327), Claudin-3 (ab15102) were purchased from Cell Signaling Technology (Danvers, MA, USA) or Abcam (Cambridge, UK). The goat anti-rabbit/goat anti-mouse secondary antibody was obtained from Boster Biological Technology (Pleasanton, CA, USA). Enzyme-linked immunosorbent assay (ELISA) kits for IL-1β (432604), IL-6 (431307), and TNF-α (430907) were purchased from Biolegend (San Diego, CA 92121, USA). Reverse transcription kits (OligdT, RRI, dNTP Mix 10 mM, MLV, MLV Buffer) and 2×SYBR Premix were obtained from Takara Biomedical Technology Co., Ltd. (Kyoto, Japan). The Reactive Oxygen Species Assay Kit (ROS Assay Kit) was purchased from Sigma–Aldrich (Saint Louis, MO, USA). The fluorescent secondary antibody was purchased from Invitrogen(Carlsbad, CA, USA).

### Animals

A total of 75 male BALB/c mice (ages 6-8 weeks and weights of 20–25 g) were obtained from the Animal Experimental Center of Jilin University. All animals were kept in a standard laboratory and fed sterile food and water. The laboratory was a room with central air that was controlled by a thermostat. The mice underwent animal experiments after a week of acclimatization. BALB/c mice were randomly divided into the following groups: untreated NT group, (2) OPD only pretreatment group (15 mg/kg), (3) LPS model group, (4) OPD pretreatment group LPS+OPD (10 mg/kg), and (5) OPD pretreatment group LPS+OPD (15 mg/kg). All animal experiments strictly followed the experimental guidelines. The *in vivo* experiments were performed in strict compliance with the protocols of the Institutional Animal Care and Use Committee of Jilin University (file no. SY20210611).

### ALI model

Mice were anesthetized prematurely and given sodium pentobarbital with a 45 mg/kg injection dose. The mice were fixed in the supine position, and unilateral nostril drops were added to the LPS solution to establish the ALI model. The dosage of LPS for each mouse was 50 μg, and LPS was dissolved in 20 μL normal saline. Then, the mice were rotated gently to evenly distribute LPS in their lungs. The mice were euthanized 12 h later, and lung tissue samples were collected.

### Administration of oxypeucedanin

Oxypeucedanin was purchased from Shanghai Yuanye Biological Co., Ltd. (China), was HPLC ≥ 98%, and was dissolved in sterile DMSO solvent. The mice were administered doses by intraperitoneal injection at 10 and 15 mg/kg concentrations. In the pretreatment groups, mice received a single dose of OPD 1 h before LPS was administered. The LPS and NT groups were treated with the same dosage of solvent. OPD was used to stimulate RAW264.7 cells *in vitro* at concentrations of 6.25 and 12.5 μM.

### Histopathological examination

Fresh lung tissue was collected and then fixed with 4% formaldehyde solution for 48 h. Next, paraffin tissue sections of lung tissue were obtained according to the routine embedding and sectioning procedures. The paraffin sections were soaked in hematoxylin and eosin in sequence, and finally, the results of H&E staining of lung tissue were obtained.

Scoring was based on the severity of alveolar and pulmonary interstitial congestion and hemorrhage, neutrophil infiltration, pulmonary interstitial edema, and hyaline membrane formation under the microscope. The lung injury score from 0 to 4 represents the following: 0 for normal, 1 for damage in the range of < 25%, 2 for damage in the range of 25-50%, 3 for damage in the range of 50-75%, and 4 for damage in the range of > 75%.

### Myeloperoxidase activity and ELISAs

For the detection of myeloperoxidase (MPO) activity, the detection method referred to in our previous study [16, 26, 27]. The lung tissues were ground by adding HEPES solution, and the supernatant solution was removed after centrifugation as MPO samples. Bronchoalveolar fluid (BALF) was obtained by the following methods: After 12 h of LPS induction, the mice were anesthetized in advance, the chest was gently opened, and the trachea was bluntly separated and exposed. (2) After cutting an inverted V-shaped opening on the trachea with surgical scissors, a catheter was carefully inserted through the incision and lavaged with a 1 mL syringe. (3) A total of 0.5 mL of sterile PBS was drawn each time, and this was repeated three times to obtain a liquid recovery rate of more than 80%. (4) The recovered solution was centrifuged at 3,000 rpm/min for 10 min at 4° C. The final supernatant was used as the ELISA test sample. The instructions for the MPO and ELISA procedures were strictly followed.

### Pulmonary capillary barrier permeability

Mice were injected with 1% Evans Blue (40 mg/kg, Sigma–Aldrich, St. Louis, MO, USA) via their tail veins. After 2 h, the mice were euthanized, the lungs were removed, and the water and blood on the lung tissue surfaces were aspirated with filter paper. An electronic balance was used to obtain the wet lung weights, and then 1 mL formamide was added for every 100 mg of lung tissue. The samples were placed in a 37° C incubator for 24 h. After that, the samples were fully reacted and centrifuged at 12,000 rpm for 30 min, and then the absorbances were measured at 620 nm by an enzyme reader. Finally, the experimental results were obtained.

### Lung wet/dry weight ratio

After the infusion of LPS for 12 h, the lung tissue (without bronchoalveolar lavage fluid) was collected, the liquid on the surface of the lung tissue was dried with filter paper, and the wet weight was recorded. Then, the samples were placed in a thermostatic oven at 70° C for 48 h and removed to measure the dry weight. The wet/dry weight (W/D) ratio was calculated to evaluate pulmonary edema.

### Immunofluorescence

The cell slides were immersed in concentrated sulfuric acid for 48 h, and sterile cell slides were obtained after high-pressure treatment. RAW264.7 cells were uniformly inoculated on the cell slides. Then, OPD was added to the medium, and after 1 h, LPS was added to stimulate the cells for an additional 12 h. On the next day, after being fixed and punched, the P65 antibody (1:100) was incubated overnight in a refrigerator at 4° C. The second antibody (1:1000) was incubated on the third day. It should be noted that the second antibody needs to be added in a dark room. Finally, the cell slides were closed after adding 4′,6-diamidino-2-phenylindole (Sigma, St. Louis, MO, USA). The paraffin tissue sections (5 μm) were fixed and perforated, and then the first antibody against Occludin or Claudin 3 (1:100) was placed into the refrigerator at 4° C overnight. The next day, the secondary antibody was added and incubated in a room that was dark and protected from light. Finally, DAPI was added, and the tissue sections were closed. A fluorescence microscope was used to obtain the experimental results.

### Cell culture and treatments

Mouse macrophage RAW264.7 cells were obtained from the China Cell Line Bank (Beijing, China). The cells were cultured in a 37° C cell incubator with a CO_2_ concentration of 5%. The cell culture medium used in the experiment was DMEM, 10% FBS was added to DMEM for the normal cell culture, a 25 cm^2^ cell culture flask was used, and trypsin (0.05%) was used for the cell digestion. In our experiment, cells were seeded in a 6-well plate overnight. When the cells were 80% confluent, they were placed in a serum-free medium and starved for 3 h. The cells were pretreated with OPD at different concentrations (6.25 and 12.5 μM) for 1 h and then stimulated with LPS (1 μg/ml) for 12 h. The serum-free medium was extracted and centrifuged at 12,000 rpm at 4° C for 15 min. The supernatant was used to detect the extracellular secretion of IL-6 and TNF-α (Biolegend, San Diego, CA, USA).

### CCK8 assay

RAW264.7 cells were seeded on 96-well plates with a cell density of approximately 65%. The next step was to add different concentrations of OPD and perform a 24 h stimulation. After that, the medium was discarded, and 10 μL CCK 8 (Saint-Bio Co., Shanghai, China) and 90 μL DMEM medium were added to each well for 2 h. Finally, the absorbance at 450 nm was measured with a microplate reader.

### Real-time PCR

Cells or tissues were added to TRIzol to lyse the cells. Total RNA was obtained by adding chloroform, isopropanol, and ethanol in sequence. cDNA was obtained from RNA by a two-step reverse transcription operation, and the reaction conditions were 70° C for 10 min, 42° C for 60 min, and 70° C for 15 min. The experiments were performed in strict accordance with the instructions (Takara, Japan). The cDNA was used as a template for qRT–PCR. The experimental conditions were 95° C for 4 min and 40 cycles of 95° C for 15 s and 60° C for 90 s. The primer sequences used in the experiment are shown in Table 1.

**Table 1:** The primer sequences of TNF-α, IL-1β, IL-6, iNOS, COX-2, and β-actin.

**Gene**	**Sequence**
β-actin	(F): 5’-GTCAGGTCATCACTATCGGCAAT-3’
	(R): 5’-AGAGGTCTTTACGGATGTCAACGT-3’
IL-6	(F): 5’-CCAGAAACCGCTATGAAGTTCC-3’
	(R): 5’-GTTGGGAGTGGTATCCTCTGTGA -3’
IL-1β	(F): 5’-GTTCCCATTAGACAACTGCACTACAG -3’
	(R): 5’-GTCGTTGCTTGGTTCTCCTTGTA -3’
TNF-α	(F): 5’-CCCCAAAGGGATGAGAAGTTC -3’
	(R): 5’-CCTCCACTTGGTGGTTTGCT -3’
iNOS	(F): 5’-GAACTGTAGCACAGCACAGGAAAT -3’
	(R): 5’-CGTACCGGATGAGCTGTGAAT -3’
COX-2	(F): 5’-CAGTTTATGTTGTCTGTCCAGAGTTTC -3’
	(R): 5’-CCAGCACTTCACCCATCAGTT -3’

### Reactive oxygen species (ROS) generation level

The cells were pretreated with OPD (12.5 μM) for 1 h and then stimulated with LPS (1 μg/ml) for 12 h. DHE (Dihydroethidium, 10 μM) was incubated with the cells for 30 min, and a fluorescence microscope was used to record the fluorescence intensity.

### Western blotting analysis

RIPA lysis buffer was added to the lung tissue and cell samples as a protein lysate. After centrifugation at 12,000 rpm at 4° C, the supernatant was obtained as the total protein extract. The concentration of total protein extract was determined by a Pierce™ BCA protein assay kit (Thermo Scientific, China). After adding SDS, the samples were divided into test samples with a concentration of 2 μg/μL. A 12% sodium dodecyl sulfate-polyacrylamide gel (SDS–PAGE gel) was prepared in advance, and then the proteins on the SDS–PAGE gel were transferred to PVDF membranes (Millipore, Darmstadt, Germany). Then, the membranes were used for electrophoresis and membrane transfer experiments. A primary antibody (1:1000) was incubated with the PVDF membrane. After 12 h at 4° C, a secondary antibody (1:5000) was incubated with another secondary antibody at room temperature for 1 h. Finally, information on the protein band exposure was obtained by using an enhanced chemiluminescence kit (Beyotime, Shanghai, China).

### Statistical analysis

All data are expressed as the mean ± SEM. The analysis and images were generated using GraphPad Prism 8 software. (La Jolla, CA, USA). One-way analysis of variance (one-way ANOVA) was used to compare the differences among groups. Survival curve differences were assessed by the Log-rank test. *p* < 0.05 was regarded as statistically significant.

### Data availability statement

The datasets used or analyzed during the current study are available from the corresponding author upon reasonable request.

## RESULTS

### Oxypeucedanin alleviated inflammation levels in LPS-induced ALI model mice and LPS-induced RAW264.7 cells

**Figure 1:**
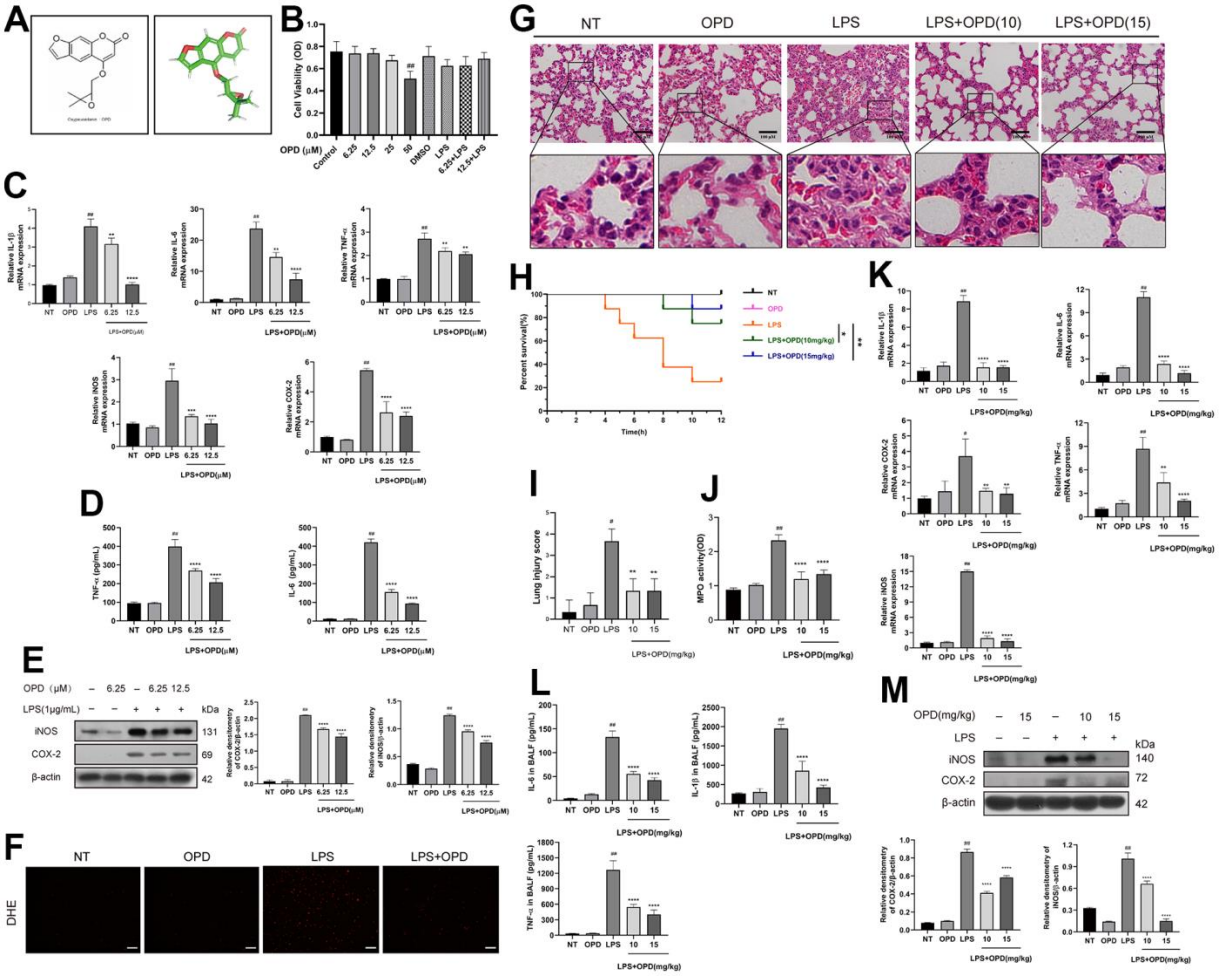
**Oxypeucedanin attenuated changes in inflammation levels in the lung tissue of LPS-induced mice and LPS-induced RAW264.7 cells.** (**A**) The molecular structure of OPD and the three-dimensional structure of the OPD molecule. (**B**) RAW264.7 cells were cultured in a 37° C cell incubator with 5% CO_2_. The cells were pretreated with OPD for 1 h, then with or without LPS (1 ug/ml) for 24 h. Different concentrations of OPD were dissolved in DMSO, and the content of DMSO was 0.1%. The effect of OPD on the viability of RAW264.7 cells was determined by the CCK8 assay. (**C**) After the OPD pretreatment for 1 h, RAW264.7 cells were stimulated with LPS (1 μg/mL) for 6 h, and total RNA was extracted by TRIzol. The mRNA transcription levels of IL-6, IL-1β, TNF-α, iNOS, and COX-2 were determined by qRT–PCR. (**D**) RAW264.7 cells were stimulated with LPS for 12 h, the culture medium was collected, and the supernatant was collected after centrifugation. IL-6 and TNF-α protein expression levels were determined by ELISA. (**E**) After 1 h of the OPD (6.25 and 12.5 μM) pretreatment, RAW264.7 cells were stimulated with LPS (1 μg/mL) for 12 h. The protein expression levels of iNOS and COX-2 were determined by Western blotting. The protein bands of iNOS and COX-2 in RAW264.7 cells are shown. The quantitative analysis of iNOS and COX-2 protein was visualized by ImageJ. (**F**) The fluorescence intensity of ROS levels was read by a fluorescence microscope; scale bar = 50 μm. (**G**) H&E staining of the lung tissue. The collected lung tissue was processed into paraffin tissue cuts and then soaked in hematoxylin and eosin in sequence, and finally, H&E staining results were obtained, scale bar = 100 μm (H&E: magnification: 200×). (**H**) After the LPS induction or equivalent volumes of saline and OPD pretreatment, the survival of mice was observed within 12 h (n=8). (**I**) The lung pathological injury score was determined as follows. Four pathological sections of H&E staining were randomly selected from each group for lung injury scoring with a 200× microscope field of view. (**J**) MPO of the lung tissue. The supernatant of fresh lung tissue was used as an MPO sample after homogenization (n=5). (**K**) The mRNA levels of inflammatory factors (IL-6, IL-1β, TNF-α, iNOS, and COX-2) in lung tissue (n=5). (**L**) The levels of IL-6, IL-1β, and TNF-α in the BALF of LPS-treated mice (n=5). (**M**) Protein levels of iNOS and COX-2 in LPS-induced mice (n=3). The quantitative analysis of iNOS and COX-2 proteins was performed by ImageJ. The concentrations of OPD in cell and animal experiments were 12.5μM and 15mg/kg, respectively. SEM was used as the error standard for data analysis, and the experiment was repeated three times independently. ^#^*p* < 0.01, ^##^*p* < 0.0001 compared with No-treatment group; ^**^p < 0.01, ^***^p < 0.001 and ^****^p < 0.0001 compared with the LPS group. LPS: Lipopolysaccharide; OPD: Oxypeucedanin; NT: No-treatment group. MPO: Myeloperoxidase LPS: Lipopolysaccharide; BALF: Bronchoalveolar Fluid.

OPD is a natural product with a molecular formula of C16H14O5 (Figure 1A). To further explore the effect of OPD on inflammation, we used RAW264.7 cells to imitate an *in vivo* model of LPS-induced inflammation. First, we completed the OPD cytotoxicity experiment on RAW264.7 cells. The results showed that OPD in the range of 25 μM had no toxic effect on the survival rate of RAW264.7 cells. However, 50 μM OPD exhibited cytotoxicity. Therefore, OPD was used at concentrations of 6.25 and 12.5 μM for the subsequent experiments (Figure 1B). The level of inflammatory mediators can reflect the degree of inflammation in RAW264.7 cells. Compared with that in the NT group, there was no difference in the levels of the IL-1β gene transcription and IL-6 and TNF-α gene transcription and protein expression in the OPD treatment only group, and there were also no differences in the gene transcription and protein expression levels of iNOS and COX-2 (Figure 1C–1E). However, LPS significantly increased the IL-1β gene transcription the gene and protein levels of IL-6 and TNF-α, and increased the gene and protein levels of iNOS and COX-2 (Figure 1C–1E). It was reassuring to note that OPD reversed the LPS-induced elevation of inflammatory mediators (Figure 1C–1E). In addition, LPS significantly increased the intracellular ROS production level, which was inhibited by an OPD pretreatment (Figure 1F). These results suggest that OPD may alleviate inflammatory injury through its intracellular ROS scavenging activity. These results indicate that inhibiting the increase in the level of proinflammatory mediators is a potential treatment for OPD to alleviate ALI.

Based on the above *in vitro* experimental results, for further in-depth study, we used LPS-induced mice to imitate the ALI model. We evaluated the effect of OPD on mouse lung tissue by H&E staining. Examining pathological tissue sections can be a good way to evaluate tissue damage. The results showed that the OPD treatment-only group did not result in damage to the lung tissue compared with that of the NT group (Figure 1G, 1I). This also demonstrated that the concentration of the drug we used was not toxic. However, LPS significantly promoted alveolar interstitial thickening, destroyed alveolar structures, and induced inflammatory cell infiltration, indicating that the ALI model was successfully constructed. Surprisingly, OPD reversed LPS-induced lung pathological tissue damage in a dose-dependent manner (Figure 1G, 1I). At the same time, we examined the survival rate of mice stimulated by LPS within 12 h. The survival rate of mice induced by LPS was significantly decreased compared to that of the NT group (Figure 1H). Oxypeucedanin improved the survival rate of mice (Figure 1H).

In ALI/ARDS, the disease is further aggravated by neutrophil infiltration, and to observe the inflammation in the lungs caused by LPS stimulation, we tested the myeloperoxidase levels. The results showed that OPD only treatment did not result in the accumulation of recruited myeloperoxidases compared with that the NT only treatment. This further demonstrates that the concentration of OPD used in this experiment is safe and nontoxic (Figure 1J). However, LPS significantly recruited the accumulation of myeloperoxidase. Surprisingly, OPD reversed the above disease phenotypes in a dose-dependent manner (Figure 1J).

The level of inflammatory mediators can reflect the degree of inflammation in lung tissues. To explore the influence of OPD on inflammatory damage and levels of inflammatory mediators in the ALI model, we tested the level of inflammatory mediators.

Similarly, LPS significantly promoted the increase in iNOS and COX-2 levels at the gene transcription and protein levels and significantly increased the expression and secretion of IL-6, IL-1β, and TNF-α at the protein and gene transcription levels (Figure 1L, 1M). It is gratifying that OPD can reverse the LPS-induced increase in the levels of iNOS, COX-2 IL-6, IL-1β, TNF-α, and other inflammatory mediators (Figure 1L, 1M); all of these results are consistent with the above *in vitro* results. These results indicate that OPD exerts a therapeutic effect on ALI by alleviating lung inflammatory injury and inhibiting the level of inflammatory mediators.

### Oxypeucedanin maintains the integrity of the lung air-blood barrier in LPS-induced mice

**Figure 2:**
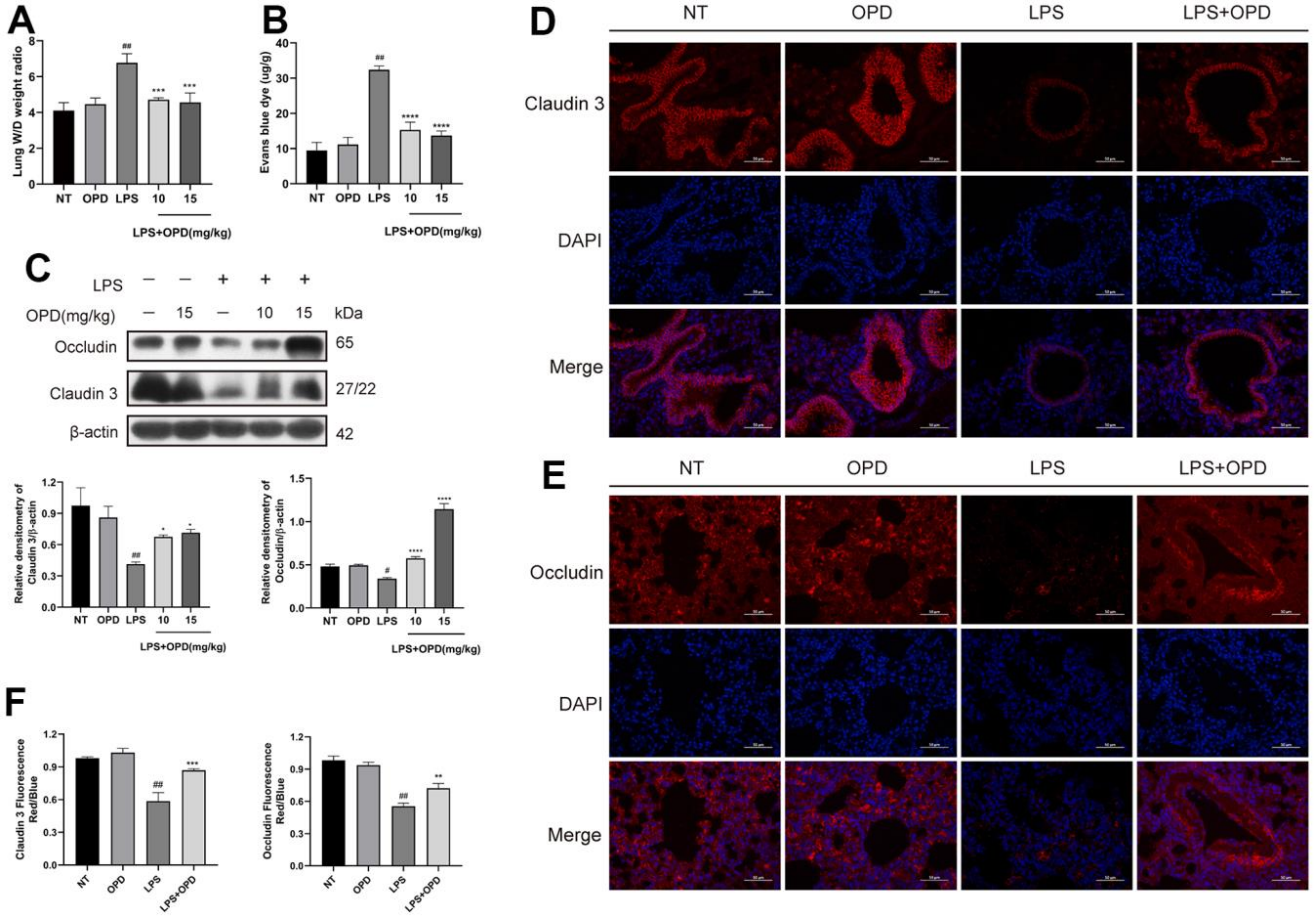
**Oxypeucedanin maintains the integrity of the lung air-blood barrier in LPS-induced mice.** LPS solution was dripped into one nostril of each mouse to establish the ALI model. Twelve hours later, a sample of mouse lung tissue was collected. OPD (10 and 15 mg/kg) was administered by intraperitoneal injection 1 h before the model was constructed. (**A**) The ratio of wet weight to dry weight of lung tissue. The lung wet/dry weight ratio was determined by the aforementioned method (n=5). (**B**) The mice were administered a tail vein injection of 1% Evans Blue (40 mg/kg, Sigma–Aldrich, MO, USA) 2 h before euthanasia. The measurement was then performed according to the method mentioned earlier. Pulmonary vascular permeability was observed by the increase in Evans blue dye in the lung tissue (n=5). (**C**) Protein levels of Occludin and Claudin 3 in LPS-treated mice (n=3). Quantitative analysis of Occludin and Claudin 3 proteins by ImageJ. (**D**–**F**) Immunofluorescence of Occludin and Claudin 3 proteins in lung tissue. Red indicates Occludin and Claudin 3 proteins, and blue indicates DAPI. Scale bar = 50 μm (n=3). Quantitative analysis of fluorescent pictures of Occludin and Claudin 3 by ImageJ. The concentration of the OPD animal experiment was 15mg/kg. SEM was used as the error standard for data analysis, and the experiment was repeated three times independently. ^#^*p* < 0.01 and ^##^*p* < 0.0001 compared with No-treatment group; ^*^p < 0.05, ^**^p < 0.01 and ^****^p < 0.0001 compared with the LPS group. LPS: Lipopolysaccharide; OPD: Oxypeucedanin; W/D: Wet/Dry weight.

To some extent, the formation of pulmonary edema reflects the increased permeability of alveolar endothelial cells and pulmonary capillaries, so we determined the lung wet/dry weight ratio. The results showed that OPD treatment alone did not cause pulmonary edema (Figure 2A). However, LPS significantly disrupted the pulmonary capillary barrier, causing pulmonary edema. Surprisingly, OPD reversed the above disease phenotypes in a dose-dependent manner (Figure 2A).

Disrupting the integrity of the pulmonary air-blood barrier causes its permeability to be altered, which further exacerbates pulmonary tissue edema and inflammatory infiltration. We used Evans blue dye to examine the lung capillary permeability, and protein expression of the tight proteins Occludin and Claudin 3 were examined by immunofluorescence and western blotting, and these proteins were observed to maintain the integrity of the pulmonary air-blood barrier.

The results showed that LPS significantly increased the pulmonary capillary permeability and significantly downregulated the tight proteins Occludin and Claudin 3 in lung tissue, but surprisingly, OPD reversed the pulmonary capillary permeability and upregulated the expression of Occludin and Claudin 3 in a concentration-dependent manner (Figure 2B, 2C). Then, immunofluorescence experiments with Occludin and Claudin 3 were completed to further verify the above results, and the results were consistent (Figure 2D–2F). These results suggest that OPD exerts a therapeutic effect on ALI by maintaining the pulmonary air-blood barrier.

### Oxypeucedanin inhibited the activation of the PI3K/AKT/NF-κB signaling pathways in LPS-induced mice and LPS-induced RAW264.7 cells

**Figure 3:**
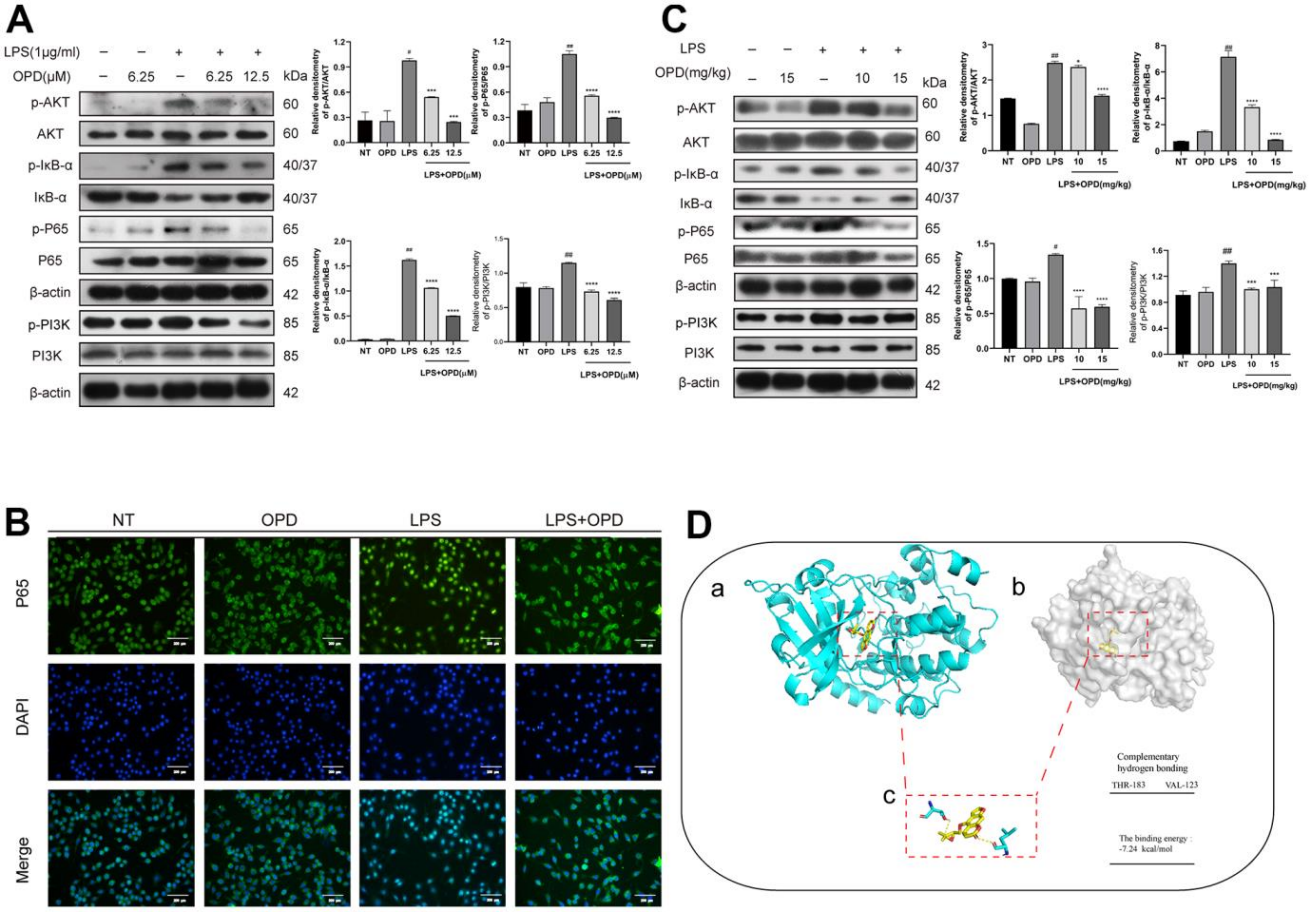
**Oxypeucedanin inhibited the activation of the PI3K/AKT/NF-κB signaling pathways in LPS-treated mice and LPS-treated RAW264.7 cells.** (**A**) The cells were pretreated with oxypeucedanin (6.25 and 12.5 μM) for 1 h and then stimulated with LPS for 1 h before the proteins were harvested. Western blotting was used to analyze the protein bands of the PI3K/AKT and NF-κB signaling pathways in RAW264.7 cells (n=5). The quantitative analysis of the protein levels in the AKT and NF-κB signaling pathways is shown. (**B**) The fluorescence intensity of the translocation of P65 into the nucleus is in RAW264.7 cells. Green indicates P65 proteins, and blue indicates DAPI. Scale bar = 200 μm (n=5). (**C**) Western blotting was used to analyze the PI3K/AKT and NF-κB signaling pathway proteins in LPS-induced mice. Quantitative analysis of the proteins in the PI3K/AKT and NF-κB signaling pathways (n=3) is shown. (**D**) The Pdb experiment database was used to download the pdb format of the AKT protein. Pubchem was used to download the sdf structure of oxypeucedanin molecules. AutoDockTools-1.5.6 and PyMOL software were used for molecular docking and data analysis. Yellow represents oxypeucedanin, and the sum can be <0 with statistical significance. (**a**, **b**) The surface binding mode of oxypeucedanin and AKT (30w3); (**c**) OPD binds to AKT (30w3) amino acid residues through hydrogen bonding. The concentrations of OPD in cell and animal experiments were 12.5μM and 15mg/kg, respectively. SEM was used as the error standard for data analysis, and the experiment was repeated three times independently. ^#^*p* < 0.01 and ^##^*p* < 0.0001 compared with No-treatment group; ^***^p< 0.001 and ^****^p < 0.0001 compared with the LPS group. LPS: Lipopolysaccharide; OPD: Oxypeucedanin; NT: No-treatment group.

We then used Western blotting and immunofluorescence technology to detect the phosphorylation degree in the PI3K/AKT/NF-κB signaling pathways and the translocation of P65 protein into the nucleus of LPS-induced RAW264.7 cells. We obtained the following experimental results: there was no difference in the phosphorylation levels of PI3K/AKT, P65, and IκB or the ubiquitination degradation levels of IκB protein between the OPD group alone and the NT group (Figure 3A). However, LPS significantly increased the phosphorylation level of AKT, P65, and IκB protein and the ubiquitination degradation level of IκB (Figure 3A, 3B). It is promising that OPD can reverse the increase in the phosphorylation levels of PI3K/AKT, P65, and IκB, which is induced by LPS, and the ubiquitination degradation level of IκB (Figure 3A).

Similarly, there was no significant difference in the level of P65 translocation into the nucleus in the OPD group alone compared with the NT group (Figure 3B). However, LPS significantly increased the probability of P65 entering the nucleus (Figure 3B). Notably, OPD reversed the nucleation level of P65 that was induced by LPS (Figure 3B). These results demonstrated that inhibiting the increase in the phosphorylation of AKT and NF-κB signaling molecules is a potential treatment for OPD to alleviate ALI.

To further verify the above results, we examined the PI3K/AKT and NF-κB signaling pathways by Western blotting in LPS-induced mice, and the results were consistent with the *in vitro* results as follows: there was no difference in the phosphorylation levels of PI3K/AKT, P65 and IκB proteins or the ubiquitinated degradation of IκB proteins in the OPD-treated group alone compared to the NT group (Figure 3C). However, LPS significantly increased the phosphorylation levels of AKT, P65, and IκB proteins as well as the ubiquitinated degradation of IκB proteins (Figure 3C). Encouragingly, OPD reversed the LPS-induced phosphorylation levels of AKT, P65, and IκB proteins and the ubiquitinated degradation of IκB proteins (Figure 3C). This result is also consistent with the results of the *in vitro* experiments described above and suggests that inhibiting the elevated levels of phosphorylation of PI3K/AKT/NF-κB signaling is a potential therapeutic means for OPD to alleviate ALI.

To determine the potential mechanism by which OPD alleviates ALI, we applied molecular biology techniques to predict the binding mode of the AKT protein and the small molecule compound OPD. The binding energy was negative, indicating that the binding effect was strong. The results showed that AKT protein created a strong bond with OPD, and the total energy was -7.24 kcal/mol (Figure 3D). In addition, THR-183 and VAL-123 of AKT are amino acid residues that form hydrogen bonds with OPD (Figure 3D). In addition, PI3K is an upstream molecule of AKT and has hydrogen bonding with OPD and the binding energy was -8.19 kcal/mol (S1). This result confirmed that PI3K/AKT is a potential target for OPD to alleviate ALI.

### Oxypeucedanin inhibited the activation of the MAPK signaling pathway in LPS-treated mice and LPS-treated RAW264.7 cells

**Figure 4:**
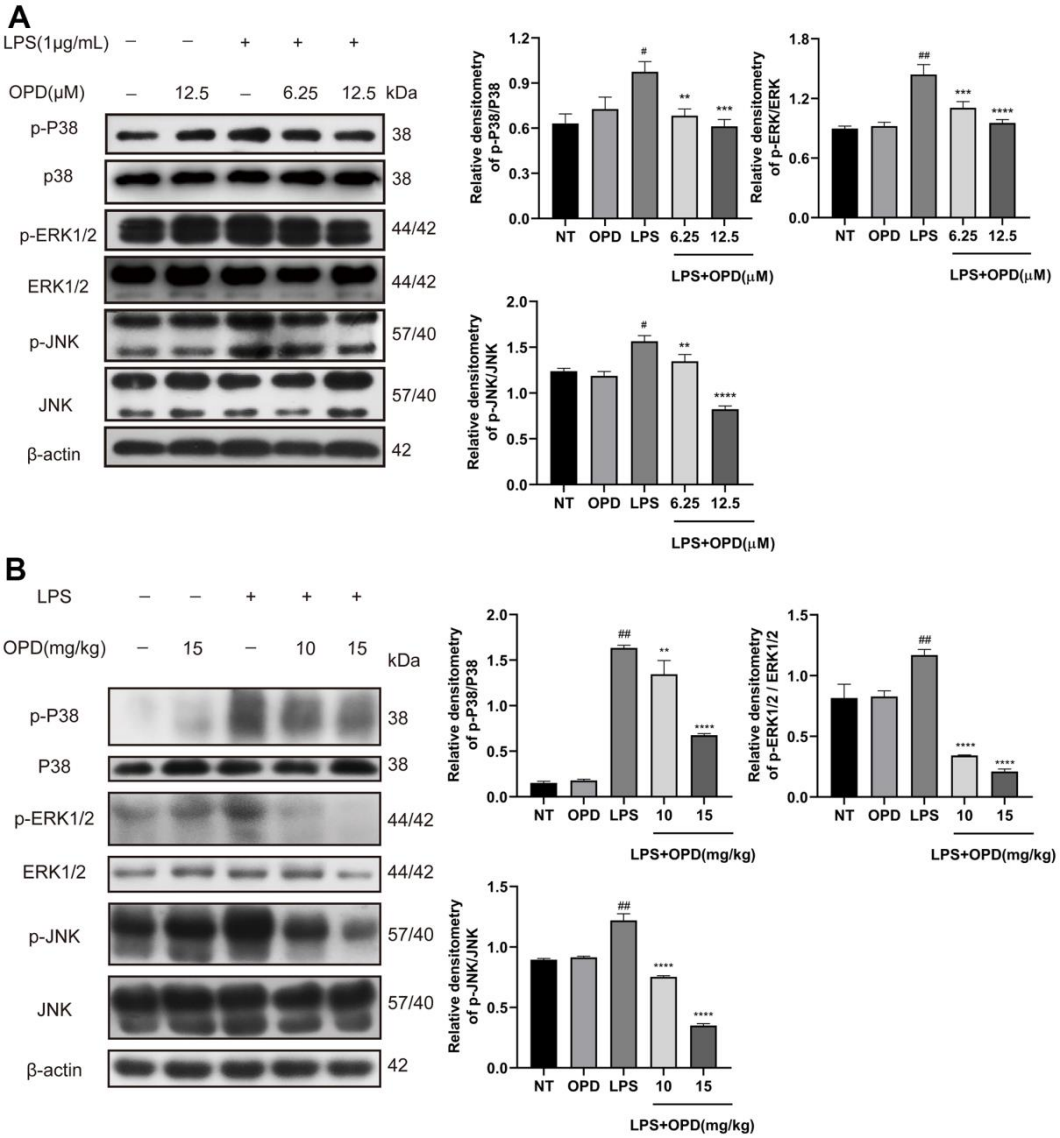
**Oxypeucedanin inhibited the activation of the MAPK signaling pathway in LPS-induced mice and LPS-induced RAW264.7 cells.** (**A**) Western blotting was used to analyze the protein of the MAPK signaling pathway in RAW264.7 cells (n=3). Quantitative analysis of the protein levels in the MAPK signaling pathway by Image J. (**B**) Western blotting was used to analyze the protein of the MAPK signaling pathway in LPS-induced mice(n=3). Quantitative analysis of protein in MAPK signaling pathway by Image J. The concentrations of OPD in cell and animal experiments were 12.5μM and 15mg/kg, respectively. SEM was used as the error standard for data analysis, and the experiment was repeated three times independently. ^#^*p* < 0.01 and ^##^*p* < 0.0001 compared with No-treatment group; ^**^p < 0.01, ^***^p < 0.001 and ^****^p < 0.0001 compared with the LPS group. LPS: Lipopolysaccharide; OPD: Oxypeucedanin; NT: No-treatment group.

The MAPK signaling pathway regulates a variety of important physiological/pathological effects, including inflammatory processes; therefore, to investigate the potential mechanism by which OPD alleviates ALI, we also applied Western blotting experimental technology to detect the MAPK signaling pathway. First, we performed the corresponding Western blotting experiment in LPS-induced RAW264.7 cells. No differences were observed in the phosphorylation levels of P38, ERK1/2, and JNK between the OPD treatment group alone and the NT group (Figure 4A). However, LPS significantly increased the phosphorylation levels of P38, ERK1/2, and JNK (Figure 4A). OPD reversed the LPS-induced increase in the phosphorylation levels of P38, ERK1/2, and JNK (Figure 4A). These results indicated that inhibiting the increase in the phosphorylation level of the MAPK signaling pathway is a potential therapeutic approach for OPD to alleviate ALI.

Then, we performed further validation tests in LPS-treated mice. Similarly, there was no difference in the phosphorylation levels of P38, ERK1/2, and JNK between the OPD treatment group alone and the NT group (Figure 4B). However, LPS significantly increased the phosphorylation levels of P38, ERK1/2, and JNK (Figure 4B). OPD reversed the LPS-induced increase in the phosphorylation levels of P38, ERK1/2, and JNK (Figure 4B). The results were also the same as the above *in vitro* experiments and further indicated that inhibiting the increase in the phosphorylation level of the MAPK signaling pathway is a potential therapeutic approach for OPD to alleviate ALI.

## DISCUSSION

ALI/ARDS is a serious respiratory disease. How to effectively alleviate ALI is a difficult problem that we urgently need to solve [1, 2]. In recent years, alleviating inflammatory diseases with natural products has become a noticeable method [28, 29]. Previous studies have shown that natural products have a good anti-pneumonia effect [19]. Therefore, a safe and efficient natural product with a low cost, low toxicity, and few side effects may be a potential therapeutic drug for alleviating ALI. Oxypeucedanin is a natural furanocoumarin compound that exists in Umbelliferae plants, such as Angelica, Imperator, Angelica dahurica, and other common Chinese medicines, and has a wide range of biological activities [22–24]. In this study, we confirmed that oxypeucedanin can adequately alleviate LPS-induced ALI in mice, and the underlying mechanism was further studied and discussed.

Pulmonary edema is a serious pathological process that occurs during ALI. On the one hand, pulmonary edema blocks the airway, causing respiratory and pulmonary ventilation and air exchange disorders and inducing local necrosis in lung tissue [2, 3, 30]. On the other hand, pulmonary edema leads to a strong inflammatory response in tissues. Large numbers of inflammatory cells are recruited, and a large number of inflammatory mediators are secreted, which aggravates tissue damage, results in the destruction of the lung air-blood barrier, and exposes the lung tissues to significant amounts of inflammation [2, 5, 31]. Therefore, relieving pulmonary edema is a potential treatment tool to alleviate ALI [5]. Studies have shown that myricetin, a natural product, can effectively alleviate the symptoms of ALI by relieving edema in lung tissue [26]. Our results demonstrated that OPD forcefully improved the ratio of wet weight to dry weight of lung tissue, which indicated that OPD relieved ALI by alleviating lung tissue edema.

The integrity of the pulmonary air-blood barrier plays a crucial role in lung physiology, and its compromised integrity leads to pulmonary edema [32]. In addition, tightly connected structures next to the endothelium and epithelium prevent vascular or interstitial fluid from infiltrating into the alveoli. In ALI, the membrane integrity of pulmonary capillaries is impaired, and pulmonary capillary permeability is increased. Our experiments showed that the OPD pretreatment reversed the LPS-induced increase in pulmonary capillary permeability. The alveolar epithelium contains tight junction structures, which play a crucial role in maintaining the integrity of the pulmonary air-blood barrier and are downregulated in ALI airway epithelium [33, 34]. It has been suggested that these tight junction proteins alleviate ALI by maintaining the integrity of the pulmonary air-blood barrier [35]. In the present study, we found that the protein expression levels of Occludin and Claudin 3 were significantly downregulated in lung tissues from the LPS group compared with the NT and OPD pretreatment groups alone; however, the OPD pretreatment significantly upregulated the protein expression levels of Occludin and Claudin 3 in the lung tissues of mice. These results suggest that the effect of OPD on LPS-induced ALI may be related to maintaining the integrity of the pulmonary air-blood barrier.

Inflammatory cytokines play a critical role in promoting inflammation. In the pathological process of ALI, the secretion of inflammatory mediators also exacerbates the severity of the disease [1, 18, 35]. The level of MPO activity reflects the degree of aggregation and activation of neutrophils within tissues. When a large number of neutrophils are activated, a respiratory burst is produced, in which a large number of oxygen free radicals and other stimulating factors are produced and damage the tissues [16]. Therefore, suppressing the activity of MPO is a method of mitigating ALI. IL-6, also known as the B cell-stimulating factor, has a wide range of biological activities and immunomodulatory effects. IL-6 mainly mediates the acute response phase of lung injury, and an abnormal increase in IL-6 can activate complement synthesis pathways for reactive proteins, and this, in turn, causes cell injury. Thus, inhibiting elevated IL-6 levels can potentially control the inflammatory response. TNF-α is a strong inflammatory mediator that acts on neutrophils to induce their respiratory bursts, which causes tissue damage. At the same time, the massive generation of TNF-α activates the NF-κB signaling pathway and thus intensifies inflammation. In addition, IL-1β plays an essential role in promoting the cascade response in the inflammatory process, so inhibiting IL-1β may be a potential means to alleviate ALI. iNOS and COX-2 are often used as strong indicators of inflammation levels, and some studies have pointed out that inhibiting iNOS and COX-2 levels may be effective in alleviating ALI. One study reported that LPS promotes proinflammatory cytokines and the recruitment of inflammatory cells. A large number of inflammatory factors lead to increased ROS production. In addition, ROS production further exacerbates the inflammatory response [36, 37]. Therefore, ROS removal may be one of the key aspects of ALI treatments. Our experimental results showed that OPD effectively alleviated pulmonary edema and lung capillary barrier injury and potently regulated the levels of inflammatory mediators, such as ROS, MPO, TNF-α, IL-1β, IL-6, iNOS, and COX-2, which may be the main reason why OPD alleviated LPS-induced ALI disease.

AKT is a protein kinase with a molecular weight of approximately 60 kDa, and PI3K/AKT is a classic signaling pathway in which the site of Ser473 of AKT is phosphorylated, which often mediates inflammation [38–40]. It has been suggested that PI3K/AKT may be a potential therapeutic target for alleviating ALI [26, 41]. NF-κB is a classical inflammatory signaling pathway, and phosphorylation of PI3K/AKT protein promotes the phosphorylation of the NF-κB signaling pathway; therefore, PI3K/AKT/NF-κB are often used as signaling axes to inhibit inflammatory diseases. P65 and IκB are the main components that constitute NF-κB, and the ubiquitinated degradation of IκB protein promotes the translocation of the P65 subunit of NF-κB into the nucleus, further leading to the secretion of large quantities of inflammatory mediators [16, 42]. High levels of inflammatory mediators are the main cause of ALI maintenance; therefore, inhibiting the activation of the PI3K/AKT/NF-κB signaling axis can effectively manipulate the inflammatory response and may be a potential therapeutic mechanism to alleviate ALI [26, 43]. Our results showed that OPD had a strong hydrogen bond with the PI3K/AKT protein, and OPD effectively inhibited the phosphorylation of the PI3K/AKT, P65, and IκB proteins and inhibited the ubiquitination and degradation of the IκB protein. Therefore, PI3K/AKT /NF-κB are potential mechanisms by which OPD alleviates ALI.

MAPKs play a key role in the regulation of inflammatory gene transcription and cytoplasmic function activity and are among the important pathways in the inflammatory signal transmission network of eukaryotes [43, 44]. Studies have shown that LPS in lung macrophages requires the involvement of MAPKs to trigger the production of pro-inflammatory enzymes and proinflammatory mediators [26, 45]. In addition, it has been pointed out that suppressing the activation of P38, ERK1/2 and JNK is a potential therapeutic strategy for alleviating ALI [43, 45, 46]. Our results also showed with certainty that OPD inhibited the phosphorylation levels of the P38, ERK1/2, and JNK proteins. The present experimental approach was consistent with the previous results when MAPKs were used as targets to alleviate ALI, and these results demonstrated that MAPKs are a potential therapeutic target for OPD to alleviate ALI.

**Figure 5:**
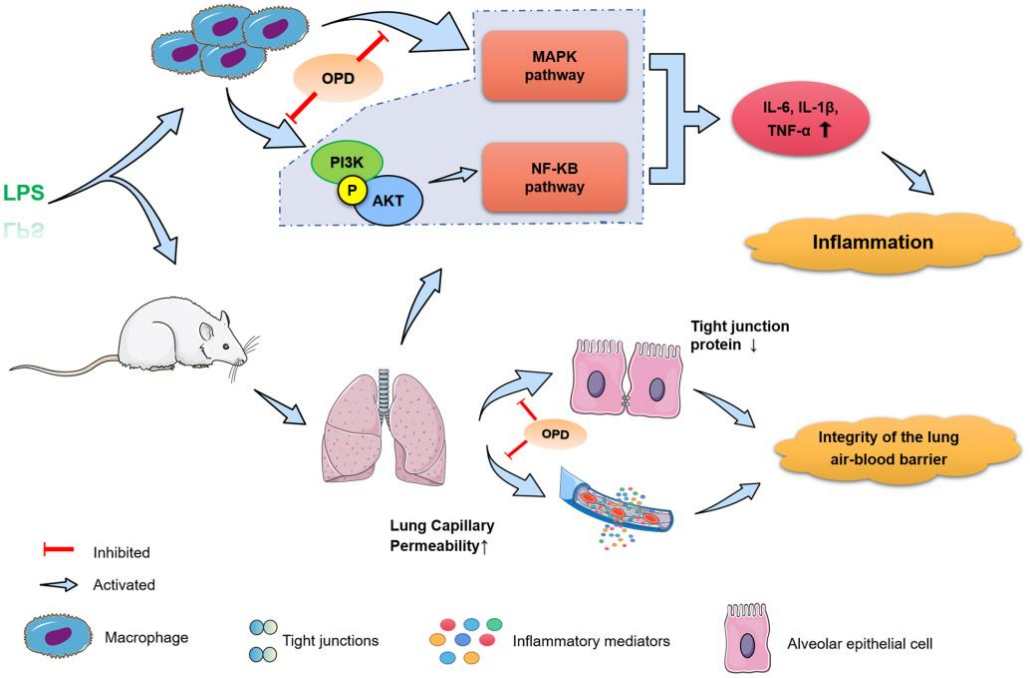
**Potential mechanism of Oxypeucedanin in alleviating acute lung injury.** OPD can significantly reduce LPS-induced acute lung injury inflammatory response and lung air-blood barrier dysfunction, and inhibited the activation of the PI3K/AKT, NF-κB, and MAPK signaling pathways, thus exerting a protective effect on acute lung injury.

Our study demonstrated the protective effect of OPD by using a model of LPS-induced inflammation *in vitro* and *in vivo*. This is an attractive direction for developing new drugs, and perhaps it may provide new options for the treatment of ALI. Based on the above experiments, the following conclusions were drawn. The therapeutic effect of OPD on ALI is mostly manifested in the effective regulation of inflammatory mediators. The potential mechanism primarily involves two aspects. OPD effectively maintained the integrity of the pulmonary air-blood barrier. On the other hand, OPD inhibited the activation of the PI3K/AKT/NF-κB and MAPK signaling pathways (Figure 5).
